# Profits of Medical Practice

**Published:** 1847-05

**Authors:** 


### THE WESTERN JOURNAL.

NEW SERIES.—VOL. VII.—NO. V.

LOUISVILLE, MAY 1, 1847.

### PROFITS OF MEDICAL PRACTICE.

That *the laborer is worthy of his hire,* is a proposition which will meet with universal assent; that the medical practitioner is one of the worst paid laborers in the land, will also, we believe, generally be conceded. While other men may decline business which pays nothing, the physician must attend to every call; and whereas the lawyer demands his fee in advance, or a note making it secure, and the mechanic expects to be paid as soon as his work is done, the doctor's bill must wait until all others are paid. A young medical friend in the interior of this State told us a short time since, that for more than a year's pretty laborious practice he had received, in cash, about twenty dollars, and he declared that if he should present his bills to his employers they would send for another physician, and he would lose his practice. Now what has brought about such a state of things? Is it not the affair of *Dan and the soaring eagle,* in the Irish legend, over again? The eagle, according to the story, caught up the napping Irishman and bore him through the fields of air in triumph; but at length his pinions grew weary and he began to complain of his burden. "*My dear fellow, who axed you?*" was Paddy's pertinent reply.

It is from no inherent weakness in their claims upon the public that physicians must thus defer them. If the practitioners of every neighborhood would agree to present their accounts at the end of the year, when all other bills are sent in, the people would soon come to feel that doctors' bills were really to be paid. We throw out this hint as one worthy of consideration. The evil complained of does not appear to us at all an incurable one, and therefore to be borne in silence. The remedy is in the hands of the practitioners themselves, and they should apply it, *tuto, cito, et jucunde.*

